# ﻿A new species of supergiant *Bathynomus* A. Milne-Edwards, 1879 (Crustacea, Isopoda, Cirolanidae) from Vietnam, with notes on the taxonomy of *Bathynomusjamesi* Kou, Chen & Li, 2017

**DOI:** 10.3897/zookeys.1223.139335

**Published:** 2025-01-14

**Authors:** Peter K. L. Ng, Conni M. Sidabalok, Thanh Son Nguyen

**Affiliations:** 1 Lee Kong Chian Natural History Museum (LKCNHM), 2 Conservatory Drive, National University of Singapore, Singapore 117377, Singapore National University of Singapore Singapore Singapore; 2 Research Center for Biosystematics and Evolution, National Research and Innovation Agency (BRIN), KST Soekarno, Jl. Raya Bogor Km 46, Cibinong 16911, Indonesia National Research and Innovation Agency Cibinong Indonesia; 3 Department of Applied Zoology, Faculty of Biology, VNU University of Science, Vietnam National University, Ha Noi, 334 Nguyen Trai, Thanh Xuan, Hanoi, Vietnam Vietnam National University Hanoi Vietnam

**Keywords:** Deep sea, fisheries, morphology, new taxon, taxonomy, seafood

## Abstract

A new supergiant species of *Bathynomus* A. Milne-Edwards, 1879 from Vietnam is described. *Bathynomusvaderi***sp. nov.** is characterised by its wide, rectangular clypeal region with parallel lateral margins, concave distal margin, and narrowly acute apex; the distally narrowing and posteriorly curved coxa of pereopod 7; and the presence of 11 upwardly curved pleotelson spines. The new *Bathynomus* is the fourth species with upwardly curved pleotelson spines and the second supergiant in the South China Sea. The taxonomy of *B.jamesi* Kou, Chen & Li, 2017 from the South China Sea is also discussed based on a large series of specimens. Previously reported differences in body form and pleotelson spines, which suggest that there may be two forms or species, are regarded as intraspecific variation for the time being. The contemporary culinary trend and fishing of *Bathynomus* in Vietnam, which have contributed to this discovery, are also discussed.

## ﻿Introduction

Four species of giant isopods of the genus *Bathynomus* A. Milne-Edwards, 1879 (Cirolanidae) are currently known from the South China Sea: *B.affinis* Richardson, 1910, *B.decemspinosus* Shih, 1972 (from southernmost Taiwan), *B.doederleini* Ortmann, 1894 (from eastern and southwestern Taiwan), and *B.jamesi* Kou, Chen & Li, 2017 (from the northern part of the South China Sea) (Shih 1972; Tso and Mok 1991; Soong and Mok 1994; Kou et al. 2017; Huang et al. 2022). The material identified as “*Bathynomuskensleyi* Lowry & Dempsey, 2006” from the South China Sea, Philippines and Hong Kong (Lowry and Dempsey 2006) and Vietnam (Truong 2015) have since been shown to be *B.jamesi*, and two individuals from near the Sulu Islands (AM P42711, AM P42712; Lowry and Dempsey 2006) appear to belong to an undescribed species (Huang et al. 2022; Huang and Bruce 2024).

Over the last seven years, *Bathynomus* has become increasingly popular in Vietnam as a delicacy in contemporary culinary culture, and it has even been compared to lobsters for the quality of the flesh (Bang 2017). The demand has resulted in increased fishing efforts to collect *Bathynomus* for the live-seafood market, and specimens have been sold alive in eateries (out of water in chilled boxes) or in cold-water tanks in large restaurants (personal observations).

As a result of the seafood trade, we managed to obtain a large series of specimens collected by the Vietnamese fishermen in Quy Nhơn City, all of which have been obtained in the South China Sea. While most of the material can be referred to *Bathynomusjamesi*, six specimens were distinct in having a differently shaped clypeal region and pleotelson structure, with the appendix masculina distinctly shorter. They are here recognised as a new species, *B.vaderi* sp. nov. and described in this paper.

## ﻿Materials and methods

Material from Vietnam were all purchased from the restaurants and local fishermen in Quy Nhơn City, Đà Nẵng City, and Hanoi. The terminology used in the description follows Bruce (1986) and Lowry and Dempsey (2006). Measurements are made of the maximum total length (TL) (in a straight line) from the base of the rostrum to the base of the pleotelson spines. Some of the drawings for description were inked electronically from a series of photographs using Adobe Illustrator v. 28.71. Specimens examined are deposited in the following institutions (with their acronyms indicated): **MNHN** – Muséum National d’Histoire Naturelle, Paris; **MZB** – Museum Zoologicum Bogoriense, BRIN, Cibinong, Indonesia; **RUMF** – Ryukyu University Museum, Fujukan, University of the Ryukyus, Japan; **TMCD** – Taiwan National Museum, Taipei, Taiwan; **ZVNU** – Zoology Collection of the Biological Museum, VNU University of Science, Hanoi, Vietnam; **ZRC** – Zoological Reference Collection of the Lee Kong Chian Natural History Museum, National University of Singapore.

## ﻿Taxonomy

**Suborder Cymothoida Wägele, 1989**

**Family Cirolanidae Dana, 1852**

### ﻿Genus Bathynomus

Taxon classificationAnimaliaIsopodaCirolanidae

A. Milne-Edwards, 1879

3E960CD5-6DEE-5177-B633-64A05FDABD9A

#### Restricted synonymy.

A. Milne-Edwards, 1879: 21—Bruce 1986: 126; Kensley and Schotte 1989: 129; Magalhães and Young 2003: 222; Lowry and Dempsey 2006: 168.

#### Type species.

*Bathynomusgiganteus* A. Milne-Edwards 1879; by monotypy.

#### Remarks.

Bruce (1986) reviewed the taxonomic history of *Bathynomus* and diagnosed the genus. Lowry and Dempsey (2006) provided the most recent comprehensive worldwide review of the *Bathynomus* species, along with their global distribution. Over the last decade, four more species have been added (Shipley et al. 2016; Kou et al. 2017; Sidabalok et al. 2020; and Huang et al. 2022), and the distributions of some have also been extended (e.g., Huang et al. 2022; Dueñas et al. 2024). To date, *Bathynomus* has 11 “supergiant” and nine “giant” extant species (Boyko et al. 2024), but there are still several species of *Bathynomus* that remain undescribed (see Sidabalok et al. 2020; Huang et al. 2022). Molecular methods (using COI and 16S rRNA sequences) have proved to be useful to help identify species as in the case of *B.jamesi*, which is morphologically similar to *B.kensleyi*, and to distinguish *B.yucatanensis* from *B.giganteus* s. str., with support from morphology (Huang et al. 2022; Huang and Bruce 2024). *Bathynomus* fossil species have been recently reviewed by Hyžný et al. (2019), with a new species described later by Hyžný et al. (2020).

### ﻿ Bathynomus jamesi

Taxon classificationAnimaliaIsopodaCirolanidae

Kou, Chen & Li, 2017

4605AB40-F938-5863-B2F8-385C952AFA2E

**Figure 1.**
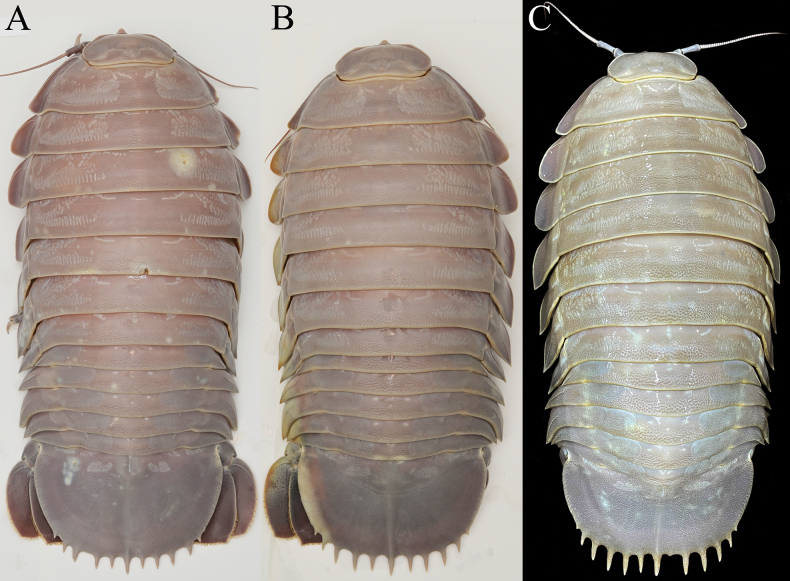
*Bathynomusjamesi* Kou, Chen & Li, 2017, colour in life, dorsal views **A** ♂ (300 mm) (ZRC 2024.0088), Vietnam **B** ♀ (280 mm) (ZRC 2024.0088), Vietnam **C** ♂ (410 mm) (ZRC 2024.0179), Vietnam.

**Figure 2.**
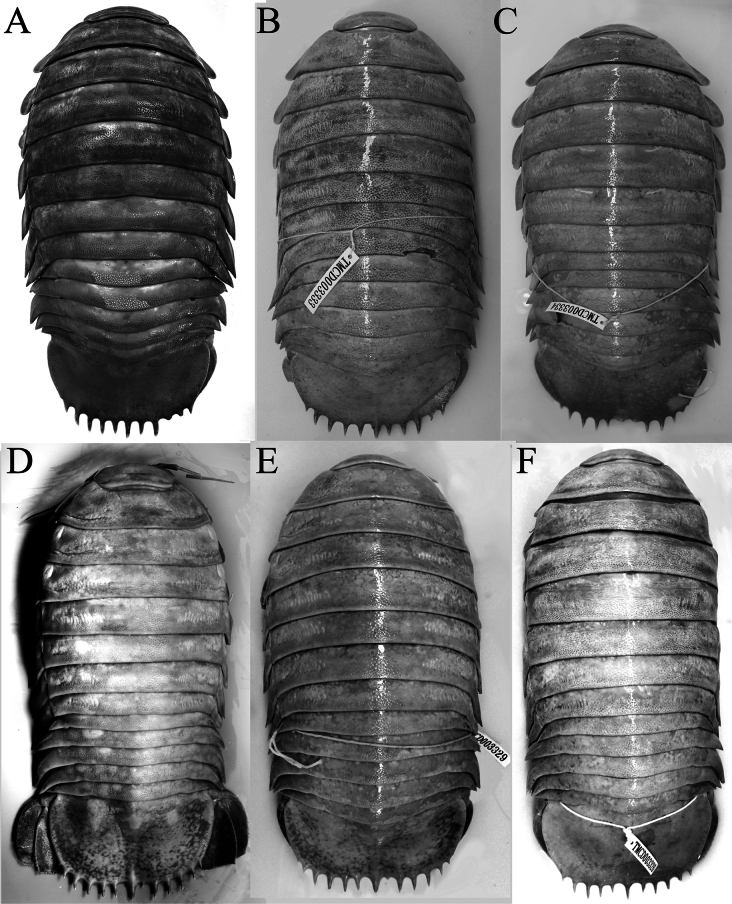
Dorsal views. *Bathynomusjamesi* Kou, Chen & Li, 2017 **A** ♂ (320 mm) (MNHN IS.2290), Philippines **B** ♂ (296 mm) (TMCD 3333), Taiwan **C** ♂ (330 mm) (TMCD 3334), Taiwan **D, E** ♀ (288 mm) (TMCD 3329), Taiwan **F** ♀ (303 mm) (TMCD 3326), Taiwan.

**Figure 3.**
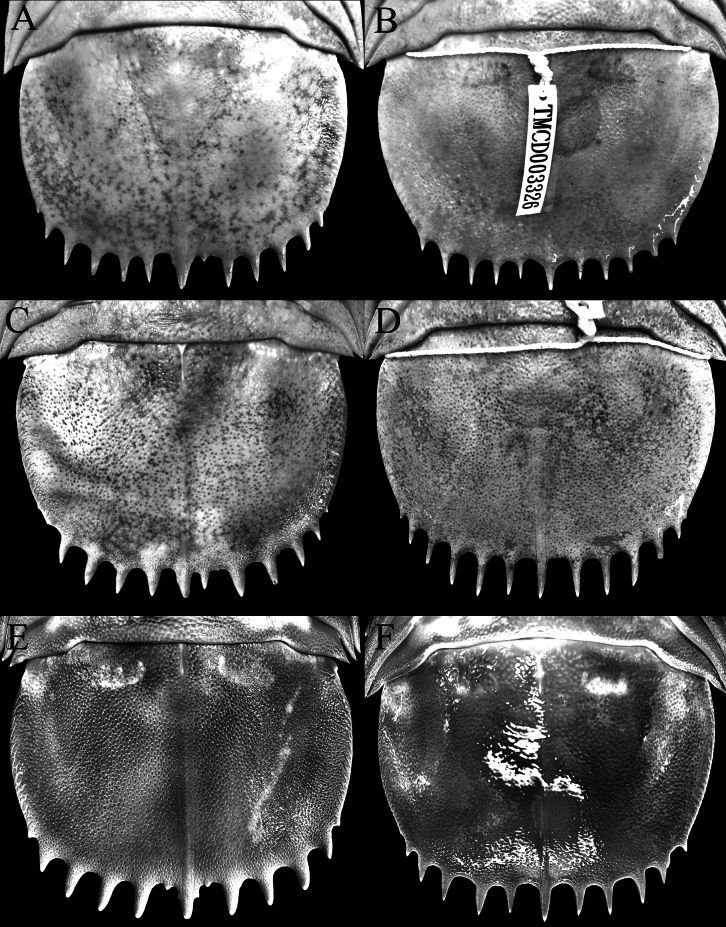
Pleotelson, dorsal views. *Bathynomusjamesi* Kou, Chen & Li, 2017 **A** ♀ (288 mm) (TMCD 3329), Taiwan **B** ♀ (303 mm) (TMCD 3326), Taiwan **C** ♂ (369 mm) (TMCD 3327), Taiwan **D** ♀ (260 mm) (TMCD 3331), Taiwan **E** ♂ (407 mm) (ZRC 2024.0118), Vietnam **F** ♀ (293 mm) (ZRC 2024.0119), Vietnam.

Figs 1 , 2 , 3 , 9E–H , 10B–D

Bathynomus kensleyi —Lowry and Dempsey 2006: 184; Truong 2015: 80. (Not Bathynomuskensleyi Lowry & Dempsey, 2006).
Bathynomus jamesi Kou, Chen & Li, 2017: 285, figs 1–5—Huang et al. 2022: 890, figs 3–8, 9a.
Bathynomus sp.—Huang et al. 2022: 902, fig. 9b.

#### Material examined.

Vietnam • 1 ♂; 300 mm; 1 ♀; 280 mm; collected by trawlers operating off Quảng Ngãi, Bình Định, Khánh Hòa and/or Phú Yên Provinces, central Vietnam; purchased by Nguyen Thanh Son from seafood markets in Hanoi; April 2024; ZRC 2024.0088 • 1 ♀; 285 mm; same collection data as for preceding; MZB. Cru. Iso 118 • 4 ♂; 415 mm, 407 mm, 380 mm, 313 mm; 1 ♀; 303 mm; same collection data as for preceding; ZRC 2024.0118 • 1 ♀; 293 mm; same collection data as for preceding; ZRC 2024.0119 • 1 ♂; 325 mm; 1 ♀; 305 mm; same collection data as for preceding; RUMF-ZC-8375 • 1 ♂; 410 mm; same collection data as for preceding; ZRC 2024.0179. Taiwan • 1 ♀; 303 mm; TMCD 3326; north part of South China Sea between North Vereker Bank (21.061°N, 116.109°E) and Pratas Island (20.717°N, 116.700°E); coll. bottom trawl, Keelung-based fishing vessel *Jin Ruiyi 37*; 17 June 2019 • 1 ♂; 369 mm; same collection data as for preceding; TMCD 3327 • 1 ♂; 314 mm; same collection data as for preceding; TMCD 3328 • 1 ♀; 288 mm; same collection data as for preceding; TMCD 3329 • 1 ♂; 342 mm; same collection data as for preceding; TMCD 3330 • 1 ♀; 260 mm; about 300 km south-west of Pratas Island (19.084°N, 115.250°E); coll. bottom trawl, Keelung-based fishing vessel *Jing Yang*; 12 May 2020; TMCD 3331 • 1 ♀; 267 mm; same collection data as for preceding; TMCD 3332 • 1 ♂; 296 mm; same collection data as for preceding; TMCD 3333 • 1 ♂; 330 mm; same collection data as for preceding; TMCD 3334. Philippines • 1 ♂; 320 mm; MUSORSTOM 2 station CP75, 13°51'N, 120°30'E, off Manila, Luzon Island 300–330 m; 25 March 1976; MNHN IS.2290.

#### Remarks.

The species was originally described from a subadult female and three juveniles by Kou et al. (2017) from off Hainan Island in the northern part of the South China Sea. Huang et al. (2022) subsequently obtained a series of specimens from Pratas (= Tungsha) Islands in the South China Sea and redescribed the species at length. Huang et al. (2022: 903) observed that there appeared to be two forms of *B.jamesi*, a slender type (with the body having the lateral edge of the pereon relatively straight; Huang et al. 2022: fig. 9b) and a stout type (with the pereonal lateral edge convex; Huang et al. 2022: fig. 9a). Huang et al. (2022) also observed that compared to the stout type, the pleotelson of the slender type was relatively longer (0.70 times as long as wide) (vs 0.42–0.56) and its pleotelson spines are flat and proximally broad (vs round and proximally narrow).

Huang et al. (2022: table 1) reported four males (TMCD 3327, 3328, 3330, 3333) and three females (TMCD 3329, 3331, 3332) of the stout type which they regarded as *B.jamesi* s. str., and one male (TMCD 3334) and one female (TMCD 3326) of the slender type which they considered as either a morphological variation of *B.jamesi* or possibly a separate species. In their photograph of the two forms, however, these authors depicted specimen TMCD 3329 as the slender type (Huang et al. 2022: fig. 9b) and TMCD 3326 as the stout type (Huang et al. 2022: fig. 9b) (sexes not stated). Their photograph of the slender type showed a proportionately longer pleotelson with the spines relatively broader and flatter while that of the stout type has a proportionately wider pleotelson with acute spines; these observations contradict what was discussed in Huang et al. (2022: 903).

We examined the specimens of Huang et al. (2022) in TMCD and found that the character states of the pereon and pleotelson discussed by them, including the associated body types in their table with codes TMCD 3326 and 3329, are indeed reversed. The slender type (based on their figured female specimen TMCD 3329) has the lateral edge of the pereon gently or distinctly convex and a proportionately wider pleotelson with the spines relatively wider and somewhat flatter (Figs 2D, E, 3A); while the stout type (based on their figured female specimen TMCD 3326) has the lateral edge of the pereon gently convex to almost straight posteriorly and a narrower pleotelson with the spines acute and more cylindrical (Figs 2F, 3B). The relative shape or convexity of the pereon and its lateral edge is not a reliable character as they are somewhat flexible; depending on how they are positioned or flexed, it can appear more slender or stout. For example, the female specimen (288 mm, TMCD 3329) figured as the slender type by Huang et al. (2022: fig. 1b) has the lateral edge of the pereon appears gently or strongly convex depending on how they are stretched and photographed (Fig. 2D, E). Other specimens from Taiwan regarded by Huang et al. (2022) as the stout type (e.g., TMCD 3327, 3333) or slender type (e.g., TMCD 3334) show varying forms of the pereon (Fig. 2B, C).

There is some variation in the kind of pleotelson spines present. Huang et al. (2022) noted that the spines may be more acute or are flattened and broader. We did not detect any pattern with the kind of spines present. The pleotelson spines do tend to be more slender, with a rounder cross-section and are usually longer in smaller specimens (ca 300 mm TL or less) (e.g., Fig. 3B, D), but we also have smaller specimens with more flattened spines as well (Fig. 3A, F). The largest specimens (exceeding 350 mm TL), however, invariably have shorter spines which are more flattened (Fig. 3C, E). We also note that the form of the median pleotelson spine also varies; in some specimens, the lateral margins have an additional tubercle, and its base may have an additional small spine or sharp tubercle (Fig. 3A, E).

For the adult specimens from Taiwan and Vietnam, the shape and proportions of the pleotelson appears to vary rather considerably, from proportionately wider and subrectangular in shape to narrower and more rounded, with length-to-width ratios ranging from 0.57–0.72 (Fig. 3). This is unexpectedly substantial for one species. As the shape of the pleotelson is a critically important and usually highly consistent species character in cirolanid taxonomy (as is the number of robust setae on the appendages), this was rather surprising. For the Taiwanese specimens examined by Huang et al. (2022), the pleotelson ratios are 0.57 and 0.71 for the two specimens of the “slender type” they reported (Fig. 3A), with the rest of the specimens (the “stout type”) ranging from 0.58–0.72 (Fig. 3B–D). As discussed above, we cannot differentiate their two types for the specimens from Taiwan and Vietnam we examined. There is, however, some correlation with size as the specimens below 300 mm TL tend to have relatively longer pleotelsons, with the ratios 0.70–0.73 (Fig. 3A, D, F). That being said, while most specimens above 300 mm TL have pleotelson ratios ranging from 0.63–0.68, the largest specimens from Vietnam exceeding 400 mm TL (ZRC 2024.0118, ZRC 2024.0179) have ratios of 0.72 as well (Fig. 3E). There is no correlation of pleotelson shape with sex. We also could not correlate pleotelson proportions with the kind of spines present along the margin. Those with more spines that have a relatively flat cross-section (Fig. 3A, C, E, F) have ratios of 0.63–0.71, while those spines that have a more rounded cross-section (Fig. 3B, D) range from 0.63–0.72.

The degree of within species variation observed in pleotelson shape and setation is slight in the Cirolanidae, and for most species in most genera, pleotelson shape is a prime taxonomic character in distinguishing species. This may not be the case for some cryptic species groups where the pleotelson is similar in form, but that reinforces the point of pleotelson uniformity. For example, the *Cirolana* ‘*parva*-group’ is a well-known species group established by Bruce (1986) for 13 taxa, with seven characters used to differentiate species (including structures of the frontal lamina, pereopod 1, pleotelson and uropods). This species group currently contains 34 similar looking species worldwide (Rodcharoen et al. 2016; Sidabalok and Bruce 2017; Jennings et al. 2020), and there remain many undescribed species. The degree of variation observed in *B.jamesi* specimens suggests that it may well be a species complex. To ascertain this, a much larger series of specimens collected from a wider geographical area will be needed, with the associated morphological and genetic studies done.

*Bathynomusjamesi* was first reported from Vietnam by Truong (2015) as “*Bathynomuskensleyi*” (cf. Huang et al. 2022; Huang and Bruce 2024). Truong (2015) based this record on a single specimen (sex not stated) measuring 260 mm collected by fishermen from the “Trường Sa area” (the Spratly Islands) in the South China Sea. The repository for the specimen is not known. As his figures do not show the clypeal region in frontal view, form of the pereopod 7 coxa or convexity of the pleotelson, so we cannot be certain of its identity, and as such, we retain it under *B.jamesi* for the time being.

*Bathynomusjamesi* is one of the largest supergiants known. The largest is believed to be *B.giganteus*, with one specimen from Brazil supposedly reaching 500 mm in length (Lowry and Dempsey 2006: 166). The two largest males of *B.jamesi* we have seen (ZRC 2024.0118, ZRC 2024.0179) measure 415 mm and 410 mm in length, respectively and weigh more than 2.6 kg each (Fig. 1C). This makes *B.jamesi* the largest known supergiant species (and largest isopod) in the Indo-West Pacific.

One female specimen examined (TMCD 3329, 288 mm) had the oostegites developed forming a brood pouch. There were about a dozen eggs inside the pouch, but the number of eggs is an underestimate as many had fallen out during collection and preservation.

### ﻿ Bathynomus vaderi sp. nov.

Taxon classificationAnimaliaIsopodaCirolanidae

E3178CAC-9400-549E-A75F-0DE102E892AF

https://zoobank.org/9021E442-A46D-45FA-A760-9F25F5A9B05F

**Figure 4.**
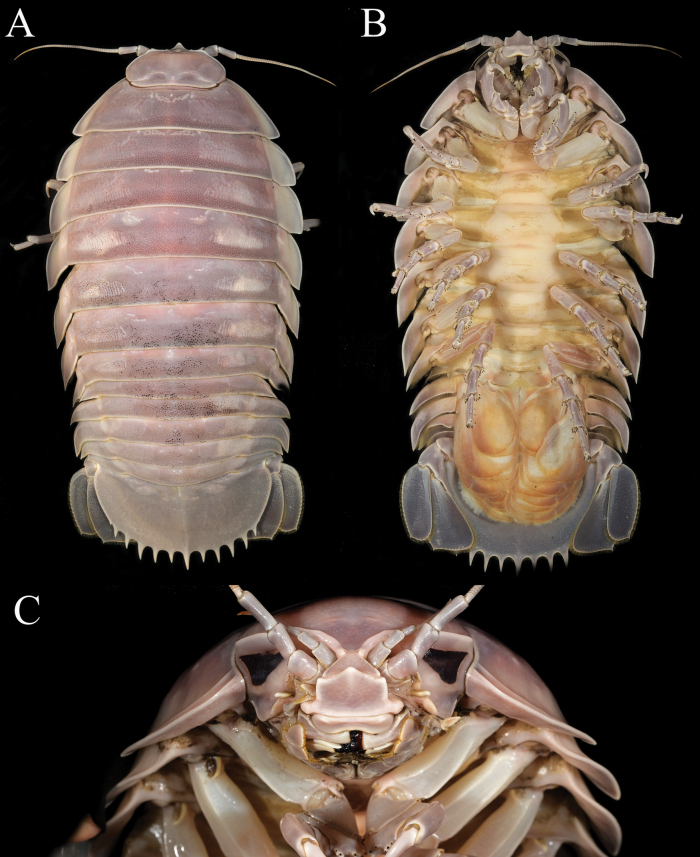
*Bathynomusvaderi* sp. nov., paratype ♂ (258 mm) (ZVNU 110001), Vietnam, colour in life **A** dorsal view **B** body, ventral view **C** cephalon, anterior view.

**Figure 5.**
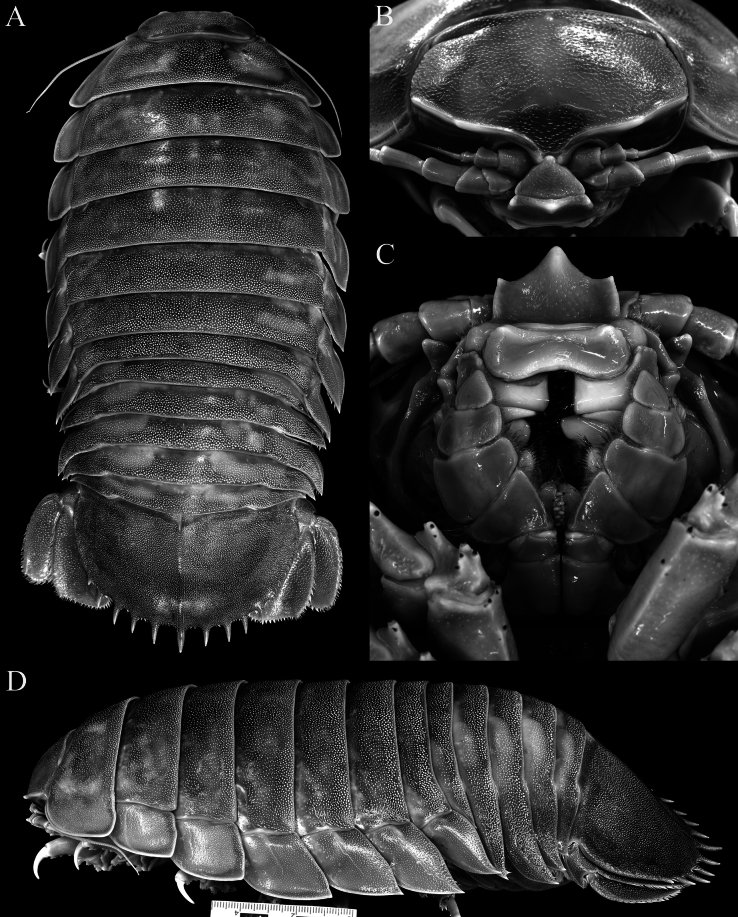
*Bathynomusvaderi* sp. nov., holotype ♂ (266 mm) (ZRC 2022.0621), Vietnam **A** dorsal view **B** cephalon, anterior view **C** clypeal region and buccal cavity **D** body, lateral view.

**Figure 6.**
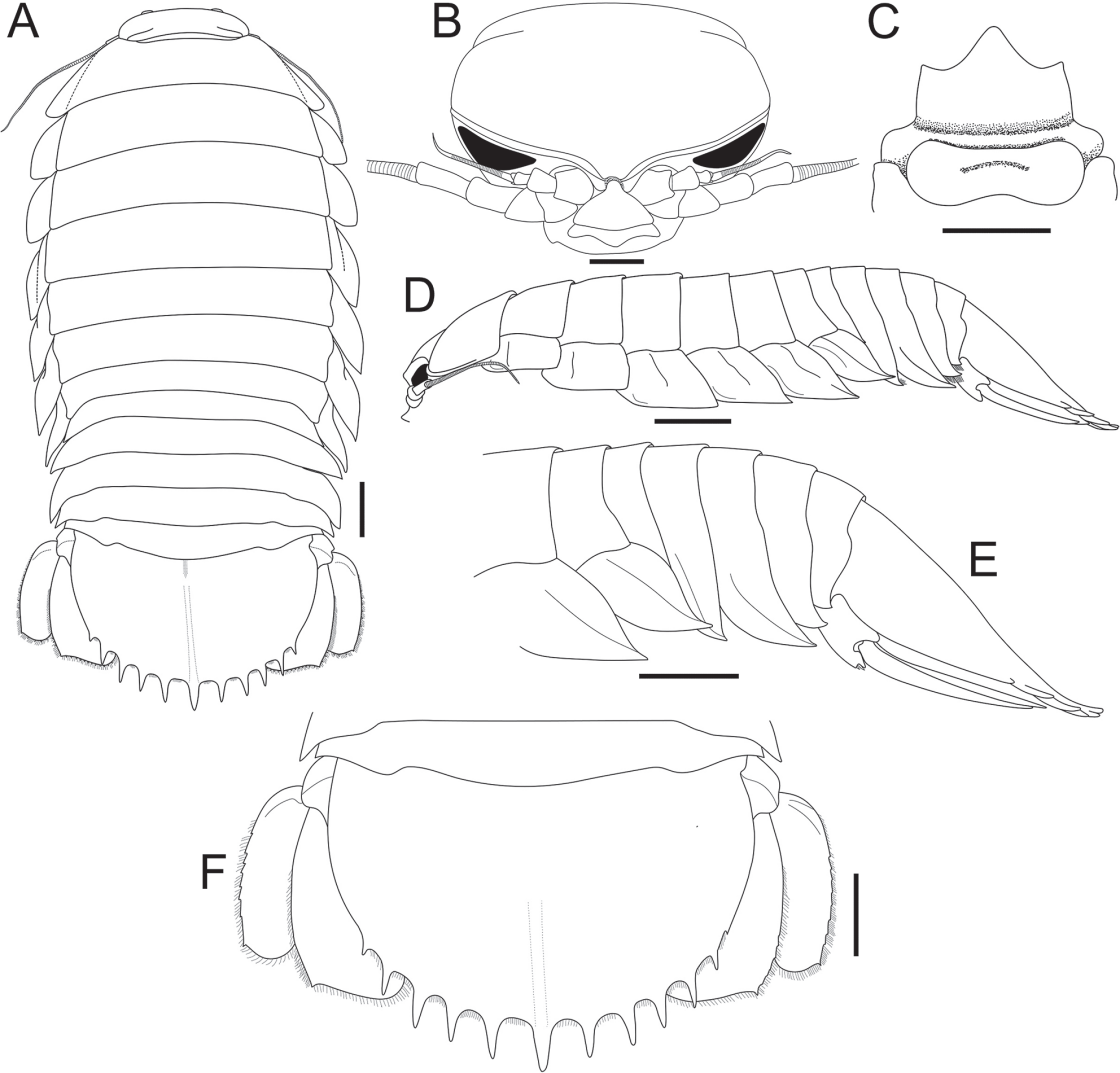
*Bathynomusvaderi* sp. nov., holotype ♂ (266 mm) (ZRC 2022.0621), Vietnam **A** dorsal view **B** cephalon, anterior view **C** clypeal region **D** body, lateral view **E** pereon, lateral view **F** pleotelson. Scale bars: 2.0 cm (**A, D–F**); 1.0 cm (**B, C**).

**Figure 7.**
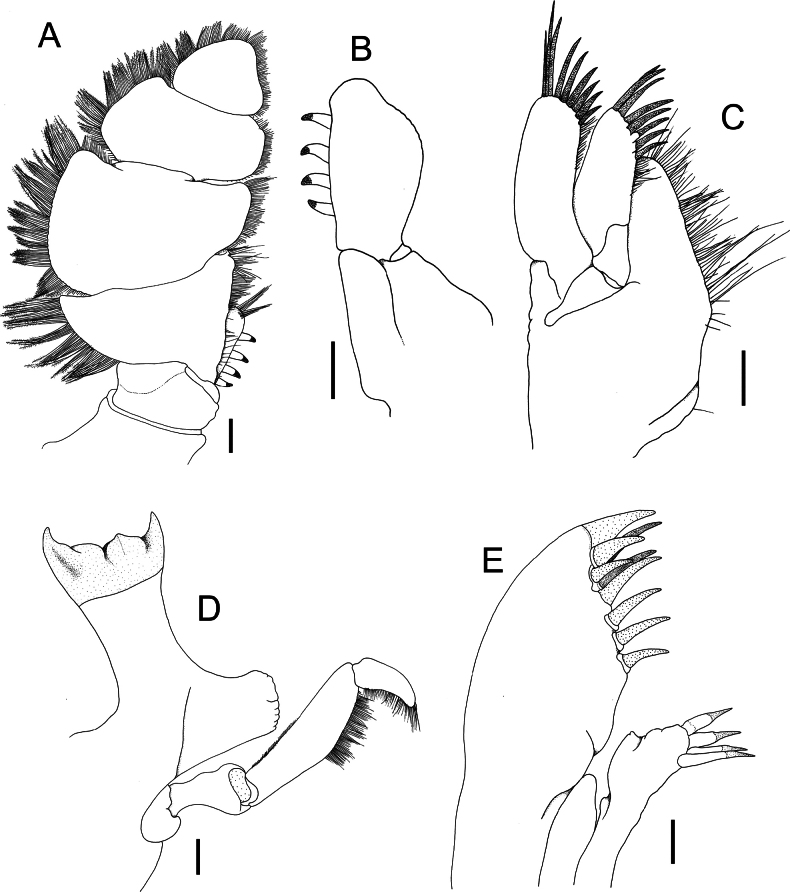
*Bathynomusvaderi* sp. nov., holotype ♂ (266 mm) (ZRC 2022.0621), Vietnam **A** right maxilliped palp, outer view **B** right maxilliped endite, inner view, setae omitted **C** right maxilla **D** left mandible and palp, outer view **E** lateral lobe of right maxilulla, outer view. Scale bars: 2.0 mm.

**Figure 8.**
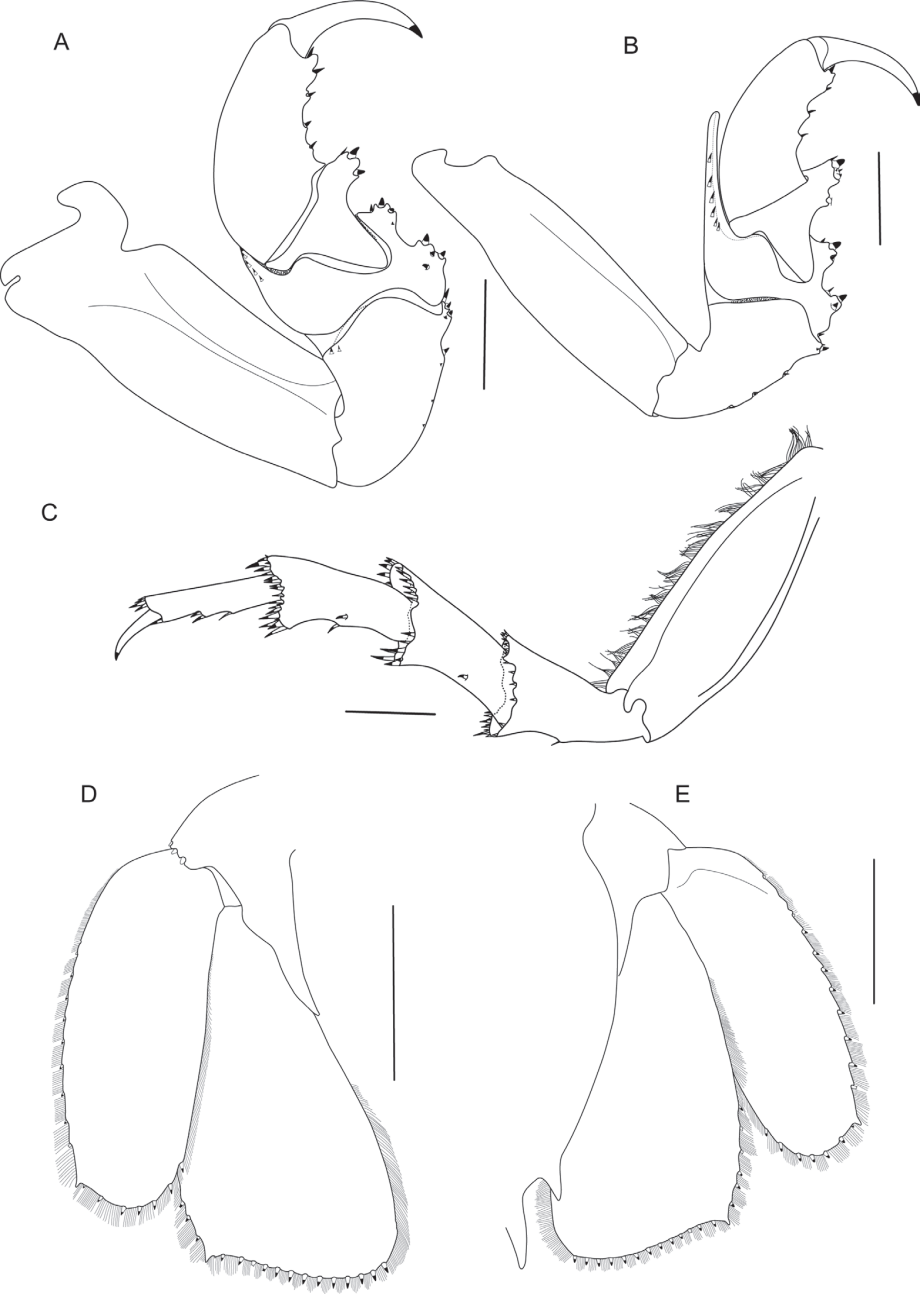
*Bathynomusvaderi* sp. nov., holotype ♂ (266 mm) (ZRC 2022.0621), Vietnam **A** pereopod 1 **B** pereopod 2 **C** pereopod 7 **D** uropod, ventral view **E** uropod, dorsal view. Scale bars: 1.0 cm (**A–C**); 2.0 cm (**D, E**).

**Figure 9.**
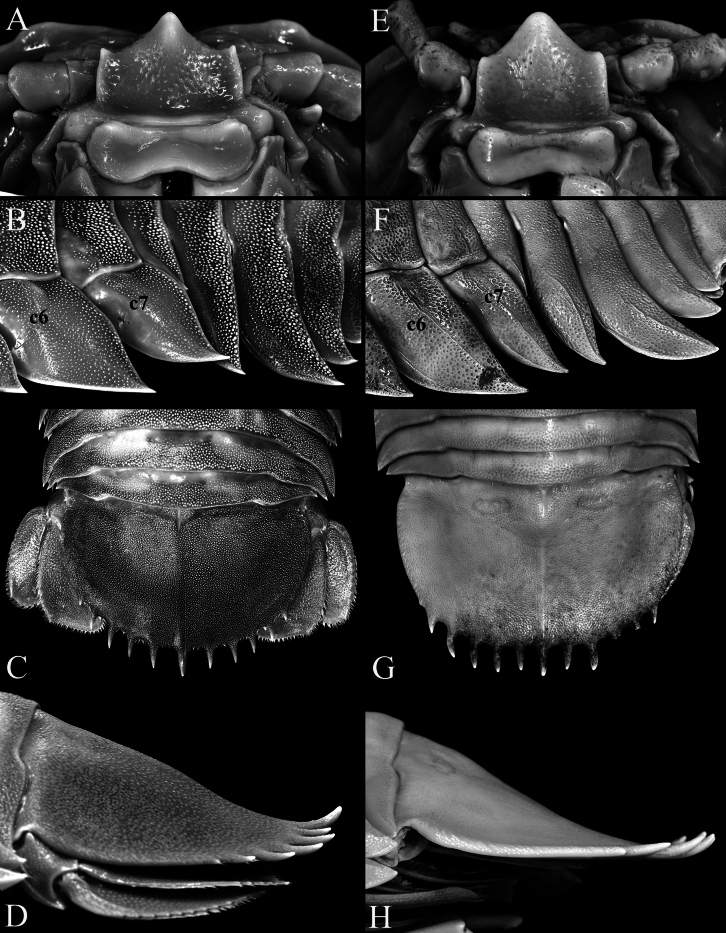
**A–D***Bathynomusvaderi* sp. nov., holotype ♂ (266 mm) (ZRC 2022.0621), Vietnam **E–H***B.jamesi* Kou, Chen & Li, 2017, ♂ (320 mm) (MNHN IS.2290), Philippines **A, E** clypeal region **B, F** pereon lateral view **C, G** pleotelson, dorsal view **D, H** pleotelson, lateral view. Abbreviations: c6 = coxa of pereopod 6; c7 = coxa of pereopod 7.

**Figure 10.**
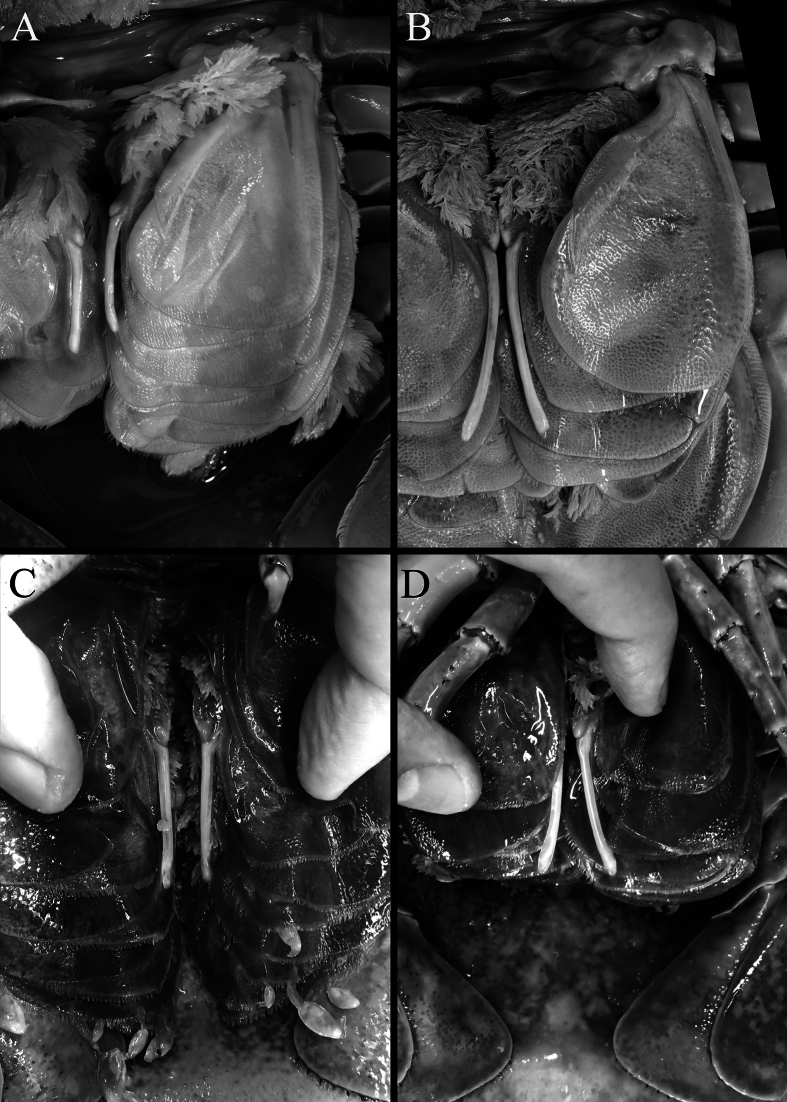
Appendix masculina **A***Bathynomusvaderi* sp. nov., paratype ♂ (270 mm) (ZRC 2024.0176) **B** ♂ *B.jamesi* Kou, Chen & Li, 2017 (320 mm) (MNHN IS.2290), Philippines **C***B.jamesi* Kou, Chen & Li, 2017 ♂ (296 mm) (TMCD 3333), Taiwan **D***B.jamesi* Kou, Chen & Li, 2017 ♂ (314 mm) (TMCD 3328), Taiwan.

Figs 4 , 5 , 6 , 7 , 8 , 9A–D , 10A

#### Material examined.

***Holotype***, Vietnam, ♂; 266 mm; offshore of Quy Nhơn City, Bình Định Province, south-central Vietnam, ca. 50 nautical miles from shore, from deep-water (depth not known); purchased by Tran Anh Duc from Eo Gió, Nhơn Lý commune; 27 March 2022; ZRC 2022.0621. ***Paratypes***: 1 ♂; 270 mm; same data as holotype; ZRC 2024.0176 • 1 ♂; 258 mm; same data as holotype; ZVNU 110001 • 1 ♂; 257 mm; same data as holotype; ZVNU 110002 • 2 ♂; 325 mm, 295 mm; collected by trawlers operating off Quảng Ngãi, Bình Định, Khánh Hòa and/or Phú Yên Provinces, central Vietnam; purchased by Nguyen Thanh Son from seafood restaurant in Đà Nẵng City, Vietnam; September 2024; ZRC 2024.0180.

#### Type locality.

Offshore of Quy Nhơn, ca 50 nautical miles from shore, south-central Vietnam, west of the Spratly Islands.

#### Diagnosis.

Clypeal region with lateral margin parallel, distal margin concave, apex narrowly acute, transversely rectangular (Figs 4C, 5C, 6C, 9A). Coxa of pereopod 7 narrows distally, curved posteriorly (Figs 5D, 6D, E, 9B). Distinct row of setae present between pleotelson spines; 11 upwardly curved pleotelson spines; pleotelson 0.6 as long as wide (Figs 5A, 9D); pleotelson vaulted laterally (Figs 6E, 9C). Appendix masculina slightly shorter than or reaching to end of endopod of pleopod 2 (Fig. 10A).

#### Description of male holotype.

Body (Figs 4A, B, 5A, 6A) 266 mm long, 135 mm wide at pereonite 5, length 1.9 times width, ovate in shape, coarsely punctate, without sculpture (Figs 4A, 5A, 6A). Head ridge above eyes discontinuous (Figs 4C, 5B, 6B). Clypeal region transversely rectangular, lateral margins parallel, distal margin slightly concave, apex narrowly subacute (Figs 4C, 5C, 6B, C, 9A).

Antennula (Fig. 6B); flagellum 1.2 longer than peduncle. Antenna peduncle article 4-articulate (Fig. 6B), article 4 about 1.4 times longer than article 3 (Fig. 6B), flagellum extending to within pereonite 2 (Fig. 6A, B).

Mandible (Fig. 7D) palp not reaching incisor, with setal fringe on lateral margin of distal half of article 2 and along article 3. Maxillula (Fig. 7E) mesial lobe with 4 robust setae, lateral lobe with 9 keratinised robust setae. Maxilla (Fig. 7C) lateral lobe with 9 keratinised robust setae, middle lobe with 7 keratinised robust setae, mesial lobe with fringe of long plumose setae. Maxilliped palp (Fig. 7A) typical of genus; maxilliped endite with 4 coupling hooks (Fig. 7B).

Pereopod 1 (Fig. 8A) ischium with 1 posteroproximal robust seta and 3 robust setae on posterodistal margin; merus with 6 robust setae on anterodistal angle, posterolateral margin with 4 robust setae in proximal row on and 3 robust setae in distal row; propodus 2.3 as long as wide, with 4 robust setae on posterior margin. Pereopod 2 (Fig. 8B) ischium with 3 robust setae on posterior margin and 4 robust setae on posterodistal margin; merus with 9 robust setae on anterodistal angle, posteromedial margin with 3 robust setae in proximal row and 3 robust setae in distal row; propodus with 4 robust setae on posterior margin. Pereopod 7 (Fig. 8C) basis 3.5 times as long as greatest width, inferior margin convex; ischium 0.5 times as long as basis, inferior margin with 1 robust seta, superior distal angle with 6 robust setae, inferior distal angle with 6 robust setae; merus 0.6 as long as ischium, as long as wide, inferior margin with 1 robust seta, superior distal angle with 11 robust setae, inferior distal angle with 13 robust setae; carpus as long as ischium, 2 times as long as wide, inferior margin with 3 robust setae (as 1 + 2), superior distal angle with 13 robust setae, inferior distal angle with 10 robust setae; propodus 0.9 as long as ischium, 3.8 times as long as wide, inferior margin with 2 clusters of robust setae (as 2 clusters of 2), superior distal angle with 8 robust setae, inferior distal angle with 1 robust seta; dactylus 0.5 as long as propodus. Coxa of pereopod 7 distally narrowed, gently curved posteriorly (Figs 5D, 6E, 9B).

Pleonite 3 (Fig. 6E) not extending beyond pleonite 5. Pleonite 4 (Fig. 6E) reaching to end pleonite 5. Penial process flat lobes (Fig. 4B). Appendix masculina with parallel margins, not extending beyond endopod, distally narrowly rounded (appendix masculina absent on holotype male) (Fig. 10A).

Pleotelson (Figs 5A, 6F, 9C, D) 0.6 time as long as greatest width, smooth (minute pores), with inconspicuous longitudinal carina on dorsal surface; dorsal surface distinctly convex (Figs 5D, 6E, 9C, D); posterior margin with 11 long, prominent, upwardly curved spines and pair of small posterolateral spines, with setae between spines, central spine simple.

Uropods (Figs 4A, 6F, 8D, E) not extending beyond pleotelson. Peduncle with 3 short robust setae on caudolateral margin (Fig. 8D); exopod and endopod with smooth lateral and distal margins; exopod lateral margin convex with 12 left and 11 right robust setae along margin, setal fringe continuous length (83.3%), medial margin straight, distomedial corner rounded, distal margin convex with 5 left and 7 right robust setae, distolateral corner produced, acute; endopod lateral margin convex, distally straight, with 5 left and 6 right robust setae; medial margin posteriorly convex; distomedial corner rounded; distal margin straight with 12 left and 13 right robust setae; distolateral corner produced, acute.

**Female.** Not known.

#### Variation.

Paratype robust setae counts as follows exopodal lateral margin with 9–13 robust setae, distal margin with 4–6, endopodal lateral margin with 1, 4 and 5 and distal margin with 10–14; pleotelson with 11 upwardly curved spines and one paratype with addition 2 small posterolateral spines. The holotype lacks the appendix masculina, but it is present in the other type specimens (Fig. 10A). As discussed by Barradas-Ortiz et al. (2003), these structures may be lost and regrow at different moults throughout the life of the animal.

#### Etymology.

The species named after the most famous Sith Lord in the *Star Wars* movie series, Darth Vader, whose helmet resembles the head of the new *Bathynomus* species.

#### Distribution.

Known only from Vietnam. We are unable to determine the exact location where *B.vaderi* was trawled, as the dealers and fishermen would only say they were obtained from deep waters off Vietnam near the Spratly Islands.

#### Remarks.

*Bathynomusvaderi* sp. nov. can be distinguished by the parallel margin of clypeal region, rectangular shape of clypeal region, the posteriorly curved coxa of pereopod 7, upwardly curved spines of the pleotelson, setae between pleotelson spines, and the laterally vaulted pleotelson. *Bathynomusvaderi* is the fourth species with upwardly curved pleotelson spines.

*Bathynomusvaderi* is very similar to the congeners with upwardly curved pleotelson spines, i.e., *B.jamesi*, *B.kensleyi*, and *B.lowryi* Bruce & Bussarawit, 2004. Other similarities with *B.jamesi* are in the length of antennae, which reaches pereonite 2, the number of pleotelson spines, pleonite 4 extending beyond pleonite 5, uropod endopod reaching the end of the pleotelson and beginning of central pleotelson spine, exopod and endopod distolateral angle is produced and subacute. *Bathynomusvaderi*, however, differs from *B.jamesi* in the following character states: the lateral margins of the clypeal region are parallel (Figs 4C, 5C, 9A) (vs gently converging distally in *B.jamesi*; Fig. 9E); the apex of the clypeal region is acute (Figs 4C, 5C, 9A) (vs obtusely rounded in *B.jamesi*; Fig. 9E); the clypeus is transversely rectangular in shape (Figs 4C, 5C, 9A) (vs square or subquadrate; Fig. 9E); the distolateral corners of the uropod endopod and exopod are acute (Fig. 8D, E) (vs subacute in *B.jamesi*; Huang et al. 2022: fig. 4d, e); the P7 coxa has the lateral margins more sinuous, with the posterior margin distinctly concave in form towards the tip (Figs 6E, 9B) (vs margins less sinuous with the posterior margin only slightly concave towards the tip in *B.jamesi*; Fig. 9F); the dorsal surface of the pleotelson is distinctly raised, being gently convex in lateral view (Figs 5D, 6D, 9D) (vs almost flat or only slightly convex in lateral view in *B.jamesi*; Fig. 9H); there are numerous short setae present between the pleotelson spines (Fig. 9C) (vs absent or only with scattered setae in *B.jamesi*; Figs 3, 9G); and the appendix masculina is shorter, just reaching to edge of endopod of pleopod 2 (Fig. 10A) (vs distinctly longer, reaching well beyond edge of endopod of pleopod 2 in *B.jamesi*; Fig. 10B–D). *Bathynomusvaderi* and *B.jamesi* are sympatric congeners in the South China Sea, a pattern of co-occurrence which has been discovered before in *B.giganteus*, *B.yucatanensis*, and *B.maxeyorum* from Gulf of Mexico (Huang et al. 2022).

The denser setation between the pleotelson spines is diagnostic for *B.vaderi* but may not be a reliable character once a larger series of specimens is collected. We note that, in *B.jamesi*, most of the specimens do not have setae or only a few scattered ones between the pleotelson spines. In a few specimens (from the recent material from Vietnam), however, the setae are slightly denser, although not to the same degree observed in *B.vaderi*.

*Bathynomusvaderi* is similar to *B.kensleyi* in the clypeal region characters, i.e., parallel margin, concave distal margin and rectangular shape; and uropod endopod characters, i.e. straight distal margin with produced acute distolateral corner. *Bathynomusvaderi* differs from *B.kensleyi* in having the clypeal region with a pointed apex (Figs 4C, 5C, 6C, 9A) (vs rounded in *B.kensleyi*; Lowry and Dempsey 2006: fig. 18E; Huang et al. 2022: fig. 2b), 11 spines on the pleotelson (Figs 4A, 5A, 6F, 9A) (vs 9 in *B.kensleyi*; Lowry and Dempsey 2006: fig. 18A, F; Huang et al. 2022: fig. 2), the pleotelson broader than long (Figs 5A, 6F, 9A) (vs longer than broad in *B.kensleyi*; Lowry and Dempsey 2006: fig. 18A, F; Huang et al. 2022: fig. 2), the uropod exopod distolateral corner produced and acute (Fig. 8D, E) (vs not produced in *B.kensleyi*; Lowry and Dempsey 2006: fig. 19D, E), and the posterior end of pleonites 3 and 4 not reaching beyond the posterior end of pleonite 5 (Figs 5D, 6E) (vs pleonite 3 exceeds pleonites 4 and 5; pleonite 4 reaches the end of pleonite 5 in *B.kensleyi*; Lowry and Dempsey 2006: fig. 18B, C; Huang et al. 2022: fig. 2a).

Other than sharing the upwardly curved pleotelson spines, there are other similarities between *B.vaderi* and *B.lowryi*: the antenna flagellum extends within pereonite 2, the clypeus is rectangular, and the pleotelson is broader than long. Both species, however, differ in having the apex of the clypeal region pointed (Figs 4C, 5C, 6C, 9A) (vs truncated in *B.lowryi*; Bruce and Bussarawit 2004: fig. 1C), 11 spines on the pleotelson (Figs 4A, 5A, 6F, 9C) (vs seven in *B.lowryi*; Bruce and Bussarawit 2004: figs 1A, 4A, 5), setae between the pleotelson spines (Figs 5A, 9C) (vs absent in *B.lowryi*; Bruce and Bussarawit 2004: figs 1A, 5), an inconspicuous longitudinal carina on the dorsal surface of the pleotelson (Figs 5A, 9C) (vs conspicuous in *B.lowryi*; Bruce and Bussarawit 2004: figs 1A, 4A, 5), a continuous setal fringe on the exopod of the uropod, which covers 83% of the margin (Fig. 8D, E) (vs fringe of medium length, covering 67% of margin in *B.lowryi*; Bruce and Bussarawit 2004: fig. 4B, C), and a convex lateral margin of the uropod exopod (Fig. 8D, E) (vs strongly convex and expanded in *B.lowryi*; Bruce and Bussarawit 2004: fig. 4B, C).

Huang et al. (2022) excluded the material from Sulu Sea previously considered to be *B.kensleyi* by Lowry and Dempsey (2006) and treated it as an undescribed species based on the states of the pereon, pleonite, maxilliped, pleotelson spines, and other characters. Based on the above characters reported by Huang et al. (2022) (species was not figured), the Sulu material is similar to *B.vaderi* in possessing more slender pleotelson spines and pleonite 4 that does not extend beyond end of pleonite 5. The two taxa, however, are different, as the Sulu Sea individual has a strongly convex uropodal exopod lateral margin (vs convex in *B.vaderi*, Fig. 8D, E). Furthermore, Shane Ahyong (pers. comm.) has compared specimens of *B.vaderi* with the Sulu Sea material, which is in the Australian Museum, and he comments that they are different taxa.

##### ﻿A note on the *Bathynomus* fishery

**Figure 11.**
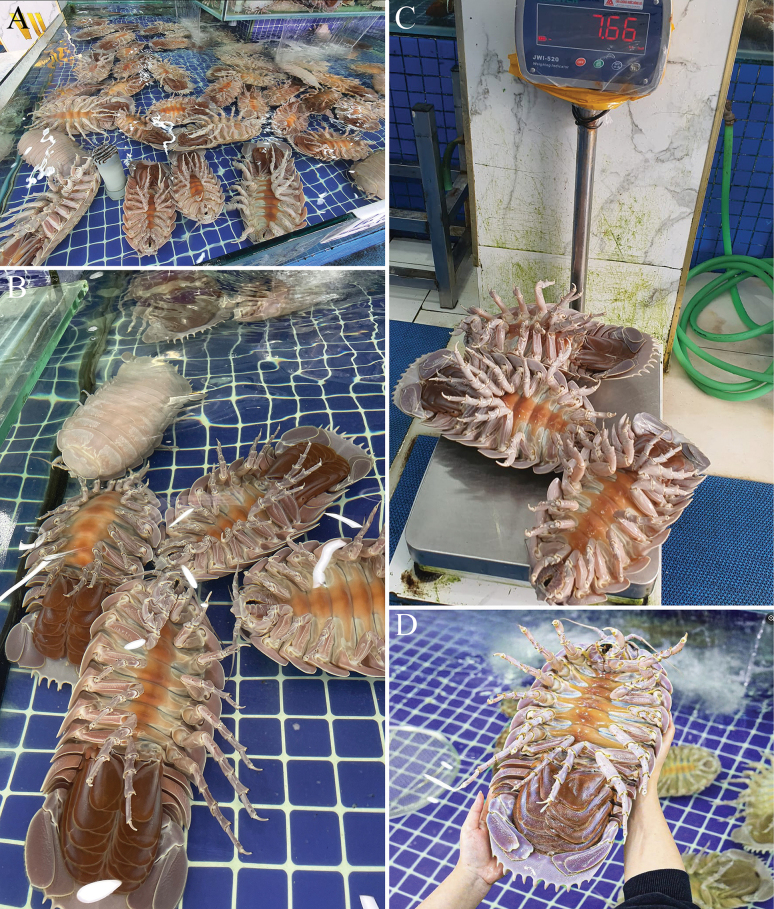
Seafood market in Hanoi, Vietnam, selling *Bathynomusjamesi***A, B** chilled water tanks keeping specimens alive for sale **C** three large specimens weighing 7.7 kg **D** Large specimens exceeding 2 kg in weight command premium prices.

In Vietnam, *Bathynomus*, known locally as bọ biển or “sea bugs”, has been fished for food apparently since 2017. Specimens are caught in deep water by trawlers operating in various parts of Biển Đông (= East Sea, Vietnamese part of the South China Sea) and brought back to shore alive in ice boxes. The isopods are kept out of water and chilled, and in this state, can survive for many days if well insulated. They are then transported to restaurants for sale. Smaller eating establishments keep the isopods in ice boxes to be cooked when asked, while large restaurants have dedicated tanks with chilled water to keep and display the animals (Fig. 11). One local restaurant owner in Eo Gió in Nhơn Lý commune (Quy Nhơn City, Bình Định Province), who was selling these isopods, explained that Bình Định, which is a coastal province at south-central Vietnam, is the main area where the isopods are caught, and a number of fishermen target these animals. Once every few days, this restaurant receives the catch from the fishermen, usually about 10 individuals each time. He also knows that they are sent alive to restaurants in Hanoi, where there is a high demand for them (Tran Anh Duc pers. comm. 2022). In Hanoi, *Bathynomus* once was sold at high prices, but this has decreased over the years. In 2017, the price was up to 2 million Vietnamese Dong (ca USD $80) per kilogram, with large individuals reaching 2 kg in weight (Bang 2017). As noted above, large specimens of *B.jamesi* can reach weights in excess of 2.5 kg. Because of these prices, fishermen started to increase the supply, and by 2023 the price dropped to around 1.5 million Vietnamese Dong per kilogram (An 2023); by early 2024, it was about 1 million Vietnamese Dong (ca USD $40) per kilogram for 1–2 kg individuals. In 2017, specimens had to be pre-ordered, and diners had to wait up to a month to collect their *Bathynomus* specimens in seafood outlets in Hanoi (Bang 2017). Today, some seafood markets in Hanoi, Hồ Chí Minh City, and Đà Nẵng City keep up to 30 individuals in their chilled water tanks for customers to buy. It is also common to see advertisements selling “sea bugs” on social network by some seafood stores. Once they are purchased online, the stores will immediately ship the alive animals in icebox to customers. Individuals weighing between 0.6–0.9 kg are the best sellers because the price is more affordable. Large specimens in excess of 2 kg are also sought after as they are less common, and their size makes for an impressive dish. In mid-2024, prices in some places in Hanoi have dropped and cost only 0.68 million Vietnamese Dong (ca USD $27) per kilogram. The prices of these animals, however, do vary quite a bit due to supply and demand, and can cost substantially more in higher end restaurants.

It is noteworthy that four type specimens of *B.vaderi* were obtained from dealers in Quy Nhơn in south-central Vietnam, where the isopods are fished. In the restaurants in Hanoi where *Bathynomus* is also sold, we have only seen *B.jamesi* so far, although we were told the specimens are also from Quy Nhơn. It is possible *B.vaderi* has a slightly different habitat, depth range, or distribution than *B.jamesi*, and what is caught depends on where individual boats trawl. In the early 2020s, *Bathynomus* was also sold for high prices in Taiwan for food, often cooked with noodles in niche restaurants (Everington 2023), with fishermen collecting them from the Pratas Islands. Fishermen collecting *Bathynomus* for restaurants in Taiwan in these islands were also the source of the material reported by Huang et al. (2022) (Huang Ming-Chih pers. comm.). During the last year, however, *Bathynomus*, is no longer popular in Taiwan, and few places sell it now (Chan Tin-Yam pers. comm.). All the supergiant specimens we know of and/or have seen from Pratas and Taiwan belong to only one species, *B.jamesi*.

## Supplementary Material

XML Treatment for Bathynomus

XML Treatment for Bathynomus jamesi

XML Treatment for Bathynomus vaderi

## ﻿Additional information

### Author ORCIDs

Peter K. L. Ng https://orcid.org/0000-0001-5946-0608

Conni M. Sidabalok https://orcid.org/0000-0002-4325-0558

Nguyen Thanh Son https://orcid.org/0000-0001-5260-5536

### Data availability

All of the data that support the findings of this study are available in the main text.
